# Supplementary material for: Direct Photochemical Synthesis of Substituted Benzo[b]fluorenes
Source: Org Lett. 2024 Nov 25;26(48):10364–8. doi: 10.1021/acs.orglett.4c03978 (PMC11629382; doi:10.1021/acs.orglett.4c03978)
Supplement: Supplementary file 2 — ol4c03978_si_002.zip [file ol4c03978_si_002.zip › FID for Publication/5m/20240531_RC-2-C-5-90B-F1_01/plots/RC-2-C-5-90B-F1_FLUORINE_20240531_01_plot01.pdf]

RC-2-C-5-90B-F1

Location	**55**	Pulse sequence	**FLUORINE**	Temperature	**25**	Suboperator	**RuairiCrawford**
Date collected	**2024-05-31**	Solvent	**cdcl3**	Spectrometer	**newucd400-vnmrs400**	Operator	**MBaumann**

SAMPLE

date	May 31 2024
solvent	cdcl3
file	/home/data/MBaumann/ucd400/RuairiCrawford/2024/20240531_RC-2-C-5-90B-F1_01/RC-2-C-5-90B-F1_FLUORINE_20240531_01.fid

ACQUISITION

sw	89285.7
at	1.000
np	178572
fb	47800
bs	2
ss	2
d1	1.000
nt	32
ct	32

TRANSMITTER

tn	F19
sfrq	376.230
tof	9882.0
tpwr	60
pw	5.000

DECOUPLER

dn	C13
dof	0
dm	nnn
decwave	W40_P8012
dpwr	34
dmf	29412

PRESATURATION

satmode	n
wet	n

SPECIAL

temp	25.0
gain	60
spin	0
hst	0.008
pw90	15.000
alfa	10.000

FLAGS

il	n
in	n
dp	y
hs	nn

PROCESSING

lb	0.50
fn	262144

DISPLAY

sp	-76625.1
wp	89285.7
rfl	76625.1
rfp	0
rp	2.9
lp	0

PLOT

wc	251
sc	8
vs	122
th	1
ai cdc ph	

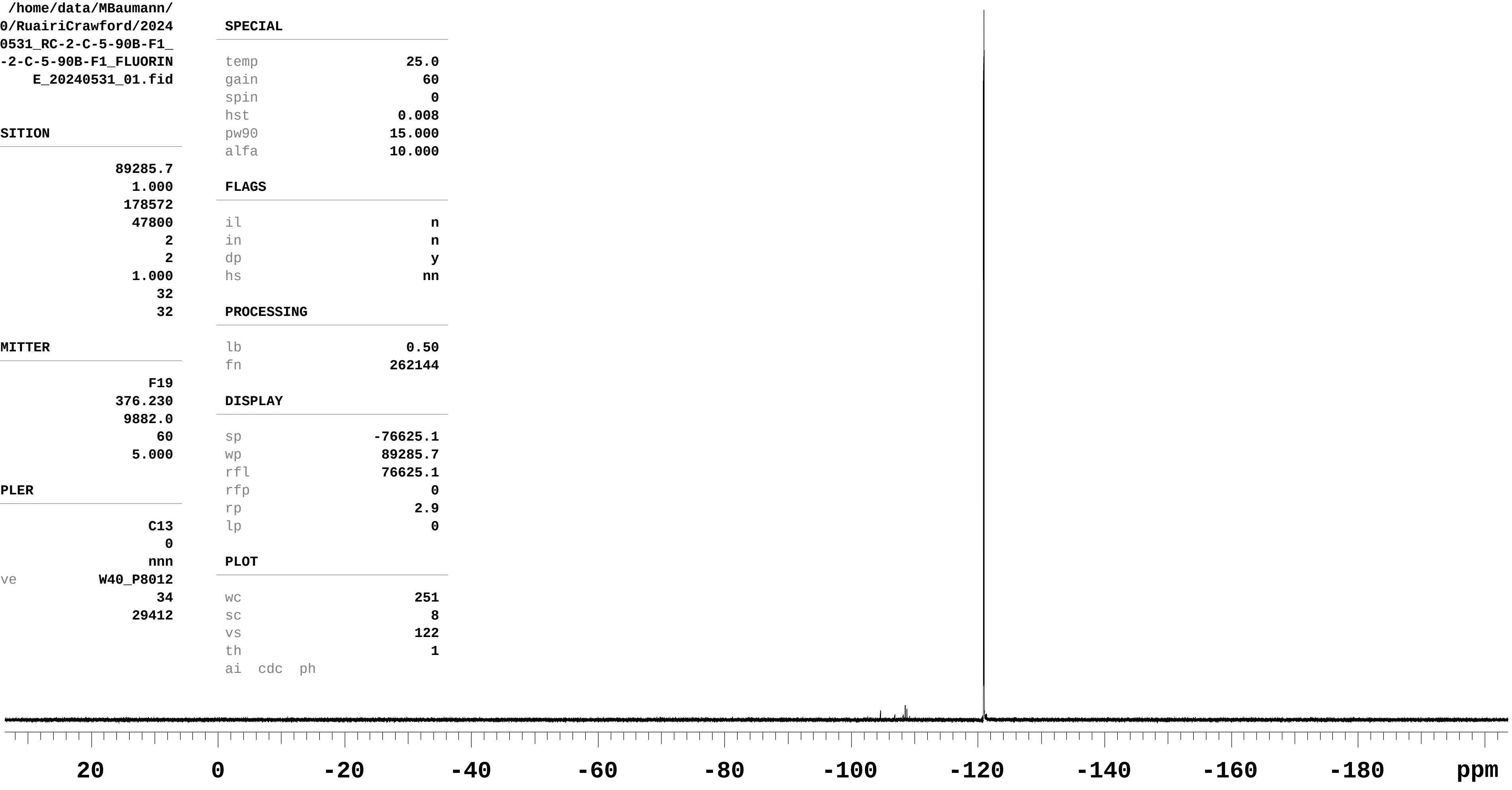

Plotname: RC-2-C-5-90B-F1_FLUORINE_20240531_01_plot01

Data file /home/data/MBaumann/ucd400/RuairiCrawford/2024/20240531_RC-2-C-5-90B-F1_01/RC-2-C-5-90B-F1_FLUORINE_20240531_01.fid

Plot date 2024-05-31
